# Efficacy and Safety of Totally Laparoscopic Gastrectomy Compared with Laparoscopic-Assisted Gastrectomy in Gastric Cancer: A Propensity Score-Weighting Analysis

**DOI:** 10.3389/fsurg.2022.868877

**Published:** 2022-05-17

**Authors:** Xin Zhong, Meng Wei, Jun Ouyang, Weibo Cao, Zewei Cheng, Yadi Huang, Yize Liang, Rudong Zhao, Wenbin Yu

**Affiliations:** Department of Gastrointestinal Surgery, General Surgery, Qilu Hospital, Cheeloo College of Medicine, Shandong University, Jinan, China

**Keywords:** totally laparoscopic gastrectomy, laparoscopic-assisted gastrectomy, laparoscopic surgery, gastric cancer, surgery prognosis

## Abstract

**Objectives:**

To compare the short- and long-term outcomes of totally laparoscopic gastrectomy (TLG) with laparoscopic-assisted gastrectomy (LAG) in gastric cancer (GC) patients and evaluate the efficacy and safety of TLG.

**Methods:**

This retrospective study was based on GC patients who underwent laparoscopic radical gastrectomy in the Qilu Hospital from January 2017 to December 2020. The groups’ variables were balanced by using the propensity score-based inverse probability of treatment weighting (PS-IPTW). The primary outcomes were 3-year relapse-free survival (RFS) and 3-year overall survival (OS). Postoperative recovery and complications were the secondary outcomes.

**Results:**

A total of 250 GC patients were included in the study. There were no significant differences in baseline and pathological features between the TLG and the LAG groups after the PS-IPTW. TLG took around 30 min longer than LAG, while there were more lymph nodes obtained and less blood loss throughout the procedure. TLG patients had less wound discomfort than LAG patients in terms of short-term prognosis. There were no significant differences between groups in the 3-year RFS rate [LAG vs. TLG: 78.86% vs. 78.00%; hazard ratio (HR) = 1.14, 95% confidence interval (CI), 0.55–2.35; *p* = 0.721] and the 3-year OS rate (LAG vs. TLG: 78.17% vs. 81.48%; HR = 0.98, 95% CI, 0.42–2.27; *p* = 0.955). The lymph node staging was found to be an independent risk factor for tumor recurrence and mortality in GC patients with laparoscopic surgery. The subgroup analysis revealed similar results of longer operation time, less blood loss, and wound discomfort in totally laparoscopic distal gastrectomy, while the totally laparoscopic total gastrectomy showed benefit only in terms of blood loss.

**Conclusion:**

TLG is effective and safe in terms of short- and long-term outcomes, with well-obtained lymph nodes, decreased intraoperative blood loss, and postoperative wound discomfort, which may be utilized as an alternative to LAG.

## Introduction

Gastric cancer is one of the most common and deadly cancers in the world, particularly in East Asia (1). Radical gastrectomy is indispensable for resectable gastric cancer (2, 3). Since Kitano et al. (4) reported the first case of laparoscopic-assisted distal gastrectomy (LADG) in 1994, laparoscopic gastrectomy has developed rapidly and been widely used.

Laparoscopic gastrectomy has two main surgical types: laparoscopic-assisted gastrectomy (LAG) and totally laparoscopic gastrectomy (TLG). The most typical procedure is LAG, which means the stomach and lymph node dissection is performed under laparoscopy, while the stomach resection and anastomosis are performed externally assisted by a 5- to 8-cm abdominal incision. Many studies have shown that there is no significant difference between LAG and open gastrectomy in the long-term prognosis for early or advanced gastric cancer (5–7). Because of the benefits of a smaller incision, less discomfort, and a speedy recovery, laparoscopic gastrectomy has increasingly become a mainstream treatment for gastric cancer (8, 9). In TLG, gastrectomy, lymph node dissection, and gastrointestinal reconstruction are performed under the laparoscopic vision, finally through an approximately 3-cm abdominal incision to take out the resection specimen. TLG eliminates the need for a large abdominal incision and provides apparent benefits in terms of exposure and anatomy (10). However, due to the lack of tactile input and the surgeon’s greater technical requirements, TLG finds it difficult to precisely define the tumor’s border and intracorporeal anastomosis.

Improvements in laparoscopic equipment, gastrointestinal reconstruction methods, and lymph node tracking technologies such as carbon nanoparticle (11) or indocyanine green (ICG) tracer-guided technologies (12, 13) are ushering in a new age of minimally invasive surgery. TLG and new intracorporeal anastomosis have attracted increased attention from scholars (14–16). However, there is currently a paucity of large-scale clinical trials to demonstrate TLG’s safety and efficacy, and the short- and long-term effects require additional medical proof to be proven.

In this study, we retrospectively analyzed TLG and LAG in gastric cancer patients by using the method of the propensity score-based inverse probability of treatment weighting (PS-IPTW) to eliminate the groups’ differences and then evaluating the short- and long-term prognoses to access the safety and effectiveness of TLG.

## Methods

### Patients

This study was based on gastric cancer patients who received laparoscopic radical gastrectomy in the department of gastrointestinal surgery of the Qilu Hospital from January 2017 to December 2020. The follow-up procedures mainly depended on the hospital’s record system and the telephone. The inclusion criteria were as follows: pathological diagnosis as gastric cancer, no history of other malignancies, and surgical methods of distal or total gastrectomy. The exclusion criteria were as follows: neoadjuvant chemotherapy, palliative surgery, distant metastasis, operation converted to laparotomy, and incomplete clinical data. The data of all patients were approved by the Ethical Review Committee of Qilu Hospital.

### Surgical Quality Control

To determine the boundary of the tumor, all TLG patients were endoscopically injected carbon nanoparticles or ICG suspension into the submucosal layer around the tumor 1 day before surgery by the same team of endoscopists. The carbon nanoparticle suspension was 0.5 mm per injection. ICG was prepared with 1.25 mg/ml sterile water and 0.5 milliliters per injection (17). All surgeries were performed by the same surgical team that had previously conducted more than 200 laparotomy and laparoscopic gastric cancer surgeries. According to the Japanese Gastric Cancer Treatment Guidelines (18), all surgeries were performed by radical gastrectomy with D2 lymph node dissection.

The patient was placed in the supine position and given general anesthesia. A subumbilical port was created and used to produce pneumoperitoneum (12–15 mmHg) (1 mmHg = 0.133 KPa). A five-port approach was used for the Trocar position. By utilizing laparoscopic exploration, the gastric resection range and digestive tract rebuilding could be determined. The upper margin should be kept at least 3–5 cm away from the cancer’s edge, while the esophageal junction cancer should be kept as far away from the cancer as feasible when enough room is conserved for esophagojejunal anastomosis, and fast-frozen pathology should be conducted when necessary.

For totally laparoscopic distal gastrectomy (TLDG), the reconstruct method was Billroth-II with Braun anastomosis. After the dissection of gastric lymph nodes, the duodenum and the distal stomach were separated with a linear closure device. A small hole was formed on the greater curvature side of the remnant stomach and on the antimesenteric border of the jejunum, 15–20 cm from the Treitz ligament. Then, a side-to-side gastrojejunostomy was performed by using a linear stapler. The entry hole for the stapler was also closed by stapling, and the anastomosis was continuously reinforced with absorbable sutures. The Braun anastomosis was performed between the input and the output loops of jejunum at 10–15 cm from the gastrojejunum anastomosis with a linear stapler. The resection specimen was put in the endobag and extracted through a small periumbilical incision.

For totally laparoscopic total gastrectomy (TLTG), the reconstruct method was the reverse puncture device reconstruction (19, 20). Following lymph node dissection, duodenum separation, and abdominal esophagus dissociation, a small hole was made on the anterior wall of the esophagus and then a small incision was made in the upper abdomen to enter the abdominal cavity. The anvil of the esophageal stump was inserted into the residual end of the esophagus, tightened, and ligated with a purse string. The linear stapler was used to close the esophagus under the anvil. The main body of the tubular stapler was placed in the distal end of the jejunum. The pneumoperitoneum was then re-established, the tubular stapler was inserted into the distal jejunum, and the central rod was connected with the anvil after penetrating the intestinal wall to complete the esophagojejunal anastomosis. The process for removing the specimen was the same as above.

For the LAG group, the Billroth-II with Braun anastomosis was performed for LADG, and the Roux-en-Y reconstruction was performed for laparoscopic-assisted total gastrectomy (LATG). The surgical methods are detailed in reference (21, 22).

### Outcome Measurements

Short-term outcomes were determined as the postoperative recovery during hospitalization. The postoperative complications were defined as the Clavien–Dindo classification ≥II (23). Long-term outcomes were measured using the time from surgery to tumor recurrence (RFS) and the time from surgery to death (OS).

### Statistical Analysis

All statistical analyses of the data were performed by using the R software 4.1.0 (R Foundation for Statistical Computing, Vienna, Austria) and the SPSS software 25.0 (IBM Corporation, Armonk, NY, USA). The continuous variables were represented by a median or average depending on the normal distribution and were analyzed by using the independent *t*-test or the Mann–Whitney *U*-test. The categorical variables were represented by the frequency and its percentage of the total and were analyzed by using the Chi-square test. To make this study closely resemble a randomized clinical trial setting, the method of the PS-IPTW was employed. Multivariable logistic regression was applied to all the baseline and pathological features between the TLG and LAG groups to generate a propensity score. And using the stabilized weights to reduce variability in IPTW models. With the goal of balancing observable characteristics, each patient was weighted by the inverse probability of being in TLG vs. LAG. The univariate and multivariate Cox proportional hazards regression model was used to analyze the independent risk factors of recurrence and mortality. The Kaplan–Meier technique and the log-rank test were used to create survival curves. Statistical significance was set at *p* < 0.05.

## Result

The demographic data and tumor characteristics are shown in Table 1. This research comprised 250 of 314 gastric cancer patients who underwent laparoscopic radical gastrectomy. A total of 156 patients were divided into the LAG group, and 90 patients were divided into the TLG group. In this study, 38 of 250 patients (15.2%) obtained a fast-frozen pathology, with only 3 cases of LAG having a positive margin, and received a second resection. All surgeries completed the R0 resection. The patients’ median age of the total group was 59 (IQR 51–66) years; 190 patients (76.0%) were men, and the average BMI was 24.51 (SD 3.44) kg/m^2^. For preoperative complications such as hypertension, heart disease, diabetes, or chronic obstructive pulmonary disease, most patients were mostly combined with 0 or 1 chronic disease (90.4%) and graded as the ASA score I/II (93.2%). Advanced gastric cancer (pT1b or above) accounted for 84% of them, and half of the tumors had lymph node metastasis (52.0%).

**Table 1:** Patient demographic data and tumor characteristics.

Characteristics	Overall	Relapse	Cox analysis		Death	Cox analysis	
	*N* = 250	*N* = 48	HR (95% CI)	*p*	*N* = 39	HR (95% CI)	*p*
Operation method							
LAG	156 (62.4)	29	Ref		24	Ref	
TLG	94 (37.6)	19	0.92 (0.51–1.65)	0.392	15	0.75 (0.39–1.44)	0.770
Age (years)	59 [51, 66]	48	1.01 (0.98–1.03)	0.661	39	1.05 (0.98–1.05)	0.378
Sex							
Female	60 (24.0)	12	Ref		12	Ref	
Male	190 (76.0)	36	0.95 (0.49–1.82)	0.871	27	0.68 (0.34–1.34)	0.267
BMI (kg/m^2^)	24.51 ± 3.44	48	0.97 (0.89–1.05)		39	0.99 (0.91–1.08)	0.798
Complications							
0	154 (61.6)	28	Ref		21	Ref	
1	72 (28.8)	17	1.31 (0.72–2.39)	0.382	16	1.68 (0.88–3.22)	0.119
2	18 (7.2)	3	0.89 (0.27–2.92)	0.842	2	0.86 (0.20–3.65)	0.833
3	5 (2.0)	0	NA	0.996	0	NA	0.996
4	1 (0.4)	0	NA	0.998	0	NA	0.999
ASA score							
I	27 (10.8)	5	Ref		4	Ref	
II	206 (82.4)	39	1.00 (0.39–2.55)	0.996	32	0.99 (0.35–2.81)	0.989
III	17 (6.8)	4	1.44 (0.38–5.42)	0.586	3	1.53 (0.34–6.89)	0.581
Tumor site							
Lower	148 (59.2)	20	Ref		9	Ref	
Middle	67 (26.8)	18	2.08 (1.10–3.94)	0.024	12	1.44 (0.69–3.00)	0.325
Upper	35 (14.0)	10	2.00 (0.94–4.28)	0.073	18	1.90 (0.85–4.24)	0.117
Gastrectomy							
Distal	155 (62.0)	18	Ref		15	Ref	
Total	95 (38.0)	30	1.06 (1.61–5.18)	<0.001	24	2.58 (1.36–4.93)	0.004
Tumor size							
≤3 cm	105 (42.0)	7	Ref		6	Ref	
>3 cm	145 (58.0)	41	4.74 (2.13–10.58)	<0.001	33	4.34 (1.82–10.36)	<0.001
Grade							
Differentiated	219 (87.6)	45	Ref		36	Ref	
Undifferentiated	31 (12.4)	3	0.45 (0.14–1.45)	0.183	3	0.56 (1.17–1.82)	0.335
pT stage							
T1a	40 (16.0)	0	Ref		0	Ref	
T1b	36 (14.4)	2	NA	0.997	2	NA	1
T2	48 (19.2)	3	NA	0.997	0	NA	0.997
T3	91 (36.4)	23	NA	0.997	19	NA	0.997
T4a	35 (14.0)	20	NA	0.996	18	NA	0.997
pN stage							
N0	120 (48.0)	4	Ref		2	Ref	
N1	32 (12.8)	3	2.97 (0.67–13.27)	0.154	2	4.21 (0.59–29.90)	0.151
N2	42 (16.8)	11	8.62 (2.74–27.08)	<0.001	9	13.54 (2.92–62.67)	<0.001
N3A	29 (11.6)	16	26.02 (8.66–78.2)	<0.001	13	41.83 (9.42–185.8)	<0.001
N3B	27 (10.8)	14	27.85 (9.10–85.3)	<0.001	13	tH5 (11.89–236.6)	<0.001
pTNM stage							
I	96 (38.4)	3	Ref		2	Ref	
II	65 (26.0)	5	2.55 (0.61–10.65)	0.201	2	1.455 (0.20–10.33)	0.708
III	89 (35.6)	40	20.48 (6.32–66.38)	<0.001	35	26.68 (6.40–111.17)	<0.001

*TLG, totally laparoscopic gastrectomy; LAG, laparoscopic-assisted gastrectomy; BMI, body mass index; Complications, the number of preoperative complications such as hypertension, heart disease, diabetes, or chronic obstructive pulmonary diseases; ASA score, assessment method by the American Society of Anesthesiologists; Gastrectomy, selection of gastrectomy included distal and total gastrectomy; TNM stage, the pathological classification under the Gastric Cancer Staging AJCC 8th edition; HR, hazard ratio; NA, not applicable; CI, confidence interval.*

The results of univariate Cox analysis revealed that the tumor site, gastrectomy, tumor size, pN stage, and pTNM stage were all closely related to tumor recurrence (*p* < 0.05). While with the exception of the tumor site, similar results were shown in the patients’ overall survival (*p* < 0.05). The pN3b stage had the highest risk factors related to recurrence [OR = 27.85 (9.10–85.30), *p* < 0.001] and death [OR = 53.05 (11.89–236.60), *p* < 0.001] in GC patients. However, the operation methods did not show significant differences in patients’ long-term prognosis (*p* > 0.05).

The PS-IPTW was applied to eliminate group bias, and the results are presented in Table 2. Before the PS-IPTW, the results of the logistic analysis of operation methods revealed that there was a significant difference between LAG and TLG in terms of ASA score and tumor size (*p* < 0.05), while age, sex, and pTNM stage showed a possible trend toward significance (*p* < 0.1). After the PS-IPTW, both the Chi-square test and the logistic analysis revealed that all the baseline and pathological variables were well-matched between the two groups (*p* > 0.1). After rounding, a total of 251 GC patients were selected for this study, of which 161 patients were of LAG and 90 patients were of TLG. In the TLG group, the median age was 58 (IQR 49–65) years, 67 (74.4%) were men, and the average BMI was 24.48 (SD 3.43) kg/m^2^. Most TLG patients were combined with 0 or 1 chronic disease (92.2%) and ASA I/II (94.4%). In terms of tumor characteristics, the majority of tumors were differentiated adenocarcinoma (88.9%) and located in the lower stomach (60.0%). A total of 77 tumors (85.6%) had invaded the submucosa or deeper regions, and 49 tumors (54.4%) had metastasized to lymph nodes. Stage II and III of pTNM constituted a majority of the TLG group (60.0%).

**Table 2:** Baseline characteristics between operation method groups.

Characteristics	Before PS-IPTW			Logistic analysis		After PS-IPTW^a^			IPTW-Logistic analysis	
	LAG (*N* = 156)	TLG (*N* = 94)	*p*	OR (95% CI)	*p*	LAG (*N* = 161)	TLG (*N* = 90)	*p*	OR (95% CI)	*p*
Age (years)	59.5 [51.8, 67.0]	57.0 [49.0, 64.0]	0.091	0.98 (0.95–1.00)	0.091	57.0 [50.0, 66.0]	58.0 [49.0, 65.0]	0.999	1.00 (0.98–1.02)	0.952
Sex										
Female	32 (20.5)	28 (29.8)	0.131	Ref		43 (26.7)	23 (25.6)	0.887	Ref	
Male	124 (79.5)	66 (70.2)		0.61 (0.34–1.10)	0.097	118 (73.3)	67 (74.4)		1.06 (0.60–1.92)	0.849
BMI (kg/m^2^)	24.50 ± 3.39	24.52 ± 3.53	0.971	1.00 (0.93–1.08)	0.971	24.33 ± 3.30	24.48 ± 3.43	0.759	1.01 (0.94–1.10)	0.729
0	96 (61.5)	58 (61.7)	0.683	Ref		103 (64.0)	57 (63.3)	0.954	Ref	
1	47 (30.1)	25 (26.6)		0.88 (0.49–1.57)	0.669	45 (28.0)	26 (28.9)		1.03 (0.57–1.83)	0.932
2	10 (6.4)	8 (8.51)		1.32 (0.49–3.55)	0.576	11 (6.8)	7 (7.8)		1.17 (0.40–3.18)	0.765
3	2 (1.3)	3 (3.19)		2.48 (0.40–19.27)	0.327	2 (1.2)	1 (1.1)		1.49 (0.11–17.07)	0.735
4	1 (0.6)	0 (0.00)		NA	0.987	1 (0.6)	0 (0.0)		NA	0.984
ASA score										
I	24 (15.4)	3 (3.19)	0.001	Ref		17 (10.6)	9 (10.0)	0.984	Ref	
II	118 (75.6)	88 (93.6)		5.97 (2.00–25.66)	0.004	133 (82.6)	76 (84.4)		1.11 (0.48–2.76)	0.806
III	14 (9.0)	3 (3.19)		1.71 (0.28–10.41)	0.542	11 (6.8)	6 (6.7)		1.09 (0.29–4.02)	0.886
Tumor site										
Lower	89 (57.1)	59 (62.8)	0.463	Ref		99 (61.5)	54 (60.0)	0.964	Ref	
Middle	42 (26.9)	25 (26.6)		0.90 (0.49–1.62)	0.723	40 (24.8)	24 (26.7)		1.10 (0.59–2.00)	0.765
Upper	25 (16.0)	10 (10.6)		0.60 (0.26–1.32)	0.218	22 (13.7)	13 (14.4)		1.06 (0.48–2.26)	0.874
Gastrectomy										
Distal	93 (59.6)	62 (66.0)	0.386	Ref		103 (64.0)	58 (64.4)	0.979	Ref	
Total	63 (40.4)	32 (34.0)		0.76 (0.44–1.29)	0.318	58 (36.0)	32 (35.6)		0.99 (0.58–1.69)	0.976
Tumor size										
≤3 cm	58 (37.2)	47 (50.0)	0.063	Ref		72 (44.7)	37 (41.1)	0.603	Ref	
>3 cm	98 (62.8)	47 (50.0)		0.59 (0.35–0.99)	0.047	89 (55.3)	54 (60.0)		1.18 (0.70–2.00)	0.536
Grade										
Differentiated	138 (88.5)	81 (86.2)	0.738	Ref		143 (88.8)	80 (88.9)	0.998	Ref	
Undifferentiated	18 (11.5)	13 (13.8)		1.23 (0.56–2.63)	0.595	18 (11.2)	10 (11.1)		1.00 (0.42–2.24)	0.998
pT stage										
T1a	24 (15.4)	16 (17.0)	0.192	Ref		23 (14.3)	13 (14.4)	0.994	Ref	
T1b	21 (13.5)	15 (16.0)		1.07 (0.43–2.69)	0.883	23 (14.3)	14 (15.6)		1.05 (0.40–2.74)	0.918
T2	24 (15.4)	24 (25.5)		1.50 (0.64–3.54)	0.349	37 (23.0)	19 (21.1)		0.92 (0.39–2.25)	0.861
T3	64 (41.0)	27 (28.7)		0.63 (0.29–1.39)	0.248	55 (34.2)	33 (36.7)		1.09 (0.48–2.45)	0.857
T4a	23 (14.7)	12 (12.8)		0.78 (0.30–2.00)	0.610	23 (14.3)	12 (13.3)		0.93 (0.34–2.47)	0.877
pN stage										
N0	70 (44.9)	50 (53.2)	0.476	Ref		80 (49.7)	41 (45.6)	0.984	Ref	
N1	22 (14.1)	10 (10.6)		0.64 (0.27–1.43)	0.286	20 (12.4)	12 (13.3)		1.12 (0.48–2.52)	0.781
N2	30 (19.2)	12 (12.8)		0.56 (0.25–1.18)	0.136	27 (16.8)	15 (16.7)		1.10 (0.52–2.27)	0.807
N3A	19 (12.2)	10 (10.6)		0.74 (0.31–1.69)	0.480	17 (10.6)	11 (12.2)		1.27 (0.54–2.90)	0.580
N3B	15 (9.6)	12 (12.8)		1.12 (0.48–2.59)	0.792	17 (10.6)	11 (12.2)		1.28 (0.54–2.94)	0.570
pTNM stage										
I	52 (33.3)	44 (46.8)	0.098	Ref		66 (41.0)	36 (40.0)	0.875	Ref	
II	45 (28.8)	20 (21.3)		0.53 (0.27–1.01)	0.057	39 (24.2)	20 (22.2)		0.95 (48–1.86)	0.880
III	59 (37.8)	30 (31.9)		0.60 (0.33–1.09)	0.094	56 (34.8)	34 (37.8)		1.15 (0.64–2.07)	0.645

*TLG, totally laparoscopic gastrectomy; LAG, laparoscopic-assisted gastrectomy; PS-IPTW, propensity score-based inverse probability of treatment weighting; ASA score, assessment method by the American Society of Anesthesiologists; TNM stage, the pathological classification under the Gastric Cancer Staging AJCC 8th edition; OR, odds ratio; CI, confidence interval*.

^a^ *Counts in the weighted cohort may not sum up to the expected totals owing to rounding. Because of rounding, percentages may not total 100, and discrepancies between numbers and percentages in the weighted cohort are the consequence of the rounding of noninteger number values.*

*The p-value was calculated by using the Chi-square test, independent t-test or Mann–Whitney U-test.

To demonstrate the high-risk factors after the PS-IPTW, the same Cox analysis procedures were used. Those significant high-risk variables before the PS-IPTW that included the tumor site, gastrectomy, tumor size, pN stage, and pTNM stage (*p* < 0.05) were reanalyzed by using univariate Cox analysis (Table 3). Following the PS-IPTW, those significant variables (*p* < 0.05) in the univariate Cox analysis were selected and included in the multivariate Cox analysis. The results showed that the pN stage was the only independent risk factor of RFS and OS in laparoscopic surgery (*p* < 0.05).

**Table 3:** Multivariate Cox analysis after PS-IPTW.

Characteristics	PS-IPTW	RFS-Cox analysis		Multi-Cox analysis		OS-Cox analysis		Multi-Cox analysis	
	*N* = 251	HR (95% CI)	*p*	HR (95% CI)	*p*	HR (95% CI)	*p*	HR (95% CI)	*p*
Tumor site									
Lower	s	Ref				Ref			
Middle	64 (25.5)	1.65 (0.78–3.49)	0.194			1.09 (0.47–2.56)	0.838		
Upper	35 (13.9)	1.74 (0.75–4.02)	0.196			1.58 (0.64–3.94)	0.322		
Gastrectomy									
Distal	161 (64.1)	Ref		Ref		Ref		Ref	
Total	91 (36.3)	2.70 (1.33–5.50)	0.006	1.00 (0.46–2.16)	0.999	2.35 (1.08–5.15)	0.032	0.66 (0.26–1.69)	0.387
Tumor size									
≤3 cm	108 (43.0)	Ref		Ref		Ref		Ref	
>3 cm	143 (57.0)	4.74 (2.13–10.58)	<0.001	1.21 (0.42–3.50)	0.729	4.08 (1.55–10.75)	0.004	0.99 (0.32–3.06)	0.984
pN stage									
N0	121 (48.2)	Ref		Ref		Ref		Ref	
N1	31 (12.4)	2.77 (0.59–13.13)	0.199	1.46 (0.21–10.11)	0.702	3.2 (0.43–24.06)	0.258	3.29 (0.71–15.20)	0.127
N2	42 (16.7)	9.08 (2.72–30.27)	<0.001	2.57 (0.44–15.00)	0.294	12.15 (2.48–59.6)	0.002	5.08 (1.48–17.45)	0.010
N3A	29 (11.6)	40.29 (12.32–131.7)	<0.001	8.91 (1.36–58.33)	0.022	59.2 (12.4–283.2)	<0.001	20.36 (4.70–88.2)	<0.001
N3B	28 (11.2)	29.84 (8.42–105.75)	<0.001	6.60 (0.97–45.03)	0.054	49.5 (9.6–254.6)	<0.001	18.30 (3.84–87.1)	<0.001
pTNM stage									
I	102 (40.6)	Ref		Ref		Ref		Ref	
II	59 (23.5)	2.73 (0.61–12.2)	0.190	1.67 (0.22–12.81)	0.620	1.29 (0.17–9.63)	0.801	0.52 (0.10–2.64)	0.434
III	90 (35.9)	26.23 (7.32–93.97)	<0.001	4.66 (0.48–45.10)	0.184	29.71 (6.5–135.73)	<0.001	3.25 (0.78–13.61)	0.106

*RFS, relapse-free survival; OS, overall survival; PS-IPTW, propensity score-based inverse probability of treatment weighting; TNM stage, the pathological classification under the Gastric Cancer Staging AJCC 8th edition; HR, hazard ratio; CI, confidence interval*.

### Operative and Prognosis Outcomes

The characteristics of operative and prognosis outcomes are presented in Table 4. Similar outcomes could be found during the PS-IPTW procedures. Following the operative outcomes, both LAG and TLG groups showed a significant difference in operation time, blood loss, and the number of lymph nodes dissected (*p* < 0.05). TLG took 30 min more than LAG (LAG vs. TLG: 240 min vs. 270 min, *p* < 0.001) but resulted in 20 ml less blood loss (LAG vs. TLG: 50 ml vs. 30 ml, *p* < 0.001). In lymph node dissection, both surgeries obtained a good number of lymph nodes (more than 16 lymph nodes), but TLG performed better (LAG vs. TLG: 28 vs. 30, *p* = 0.018).

**Table 4:** Characteristics of operative and prognosis outcomes.

Characteristics	Before PS-IPTW (*N* = 250)			After PS-IPTW (*N* = 251)		
	LAG (*N* = 156)	TLG (*N* = 94)	*p*	LAG (*N* = 161)	TLG (*N* = 90)	*p*
Operative Outcomes						
Operation time (minutes)	240.0 [210.0, 285.0]	270.0 [240.0, 300.0]	<0.001	240.0 [210.0, 282.5]	270.0 [240.0, 300.0]	<0.001
Blood loss (ml)	50.0 [40.0, 80.0]	30.00 [25.00, 40.00]	<0.001	50.0 [40.0, 90.3]	30.0 [30.0, 45.9]	<0.001
Positive LN	1.0 [0.0, 6.0]	0.0 [0.0, 6.0]	0.532	0.48 [0.00, 5.89]	1.00 [0.00, 7.00]	0.400
Dissected LN	27.0 [21.0, 35.0]	29.0 [23.0, 40.0]	0.040	28.0 [21.0, 34.0]	30.0 [24.0, 40.6]	0.018
Transfusion						
No	137 (87.82)	85 (90.43)	0.67	141 (87.6)	80 (88.9)	0.844
Yes	19 (12.18)	9 (9.57)		20 (12.4)	10 (11.1)	
Short-Term Outcomes						
First flatus (days)	3.0 [3.0, 3.0]	3.0 [2.0, 3.0]	0.486	3.0 [3.0, 3.0]	3.0 [2.0, 3.0]	0.350
First defecation (days)	4.0 [3.5, 6.0]	4.0 [3.62, 5.0]	0.831	5.0 [3.5, 5.0]	4.0 [3.50, 5.0]	0.723
First drinking water (days)	5.0 [4.0, 6.0]	5.0 [4.0, 6.0]	0.834	5.0 [4.0, 6.0]	5.0 [4.0, 7.0]	0.558
First liquid food (days)	7.0 [6.0, 8.0]	7.0 [7.0, 9.0]	0.338	7.0 [6.0, 8.0]	8.0 [7.0, 9.0]	0.177
Nasogastric tube (days)	4.0 [3.0, 6.0]	5.0 [4.0, 6.0]	0.264	4.0 [3.0, 6.0]	5.0 [4.0, 6.0]	0.168
Pain scores (points)	2.6 [2.2, 2.8]	2.2 [2.0, 2.6]	<0.001	2.6 [2.2, 2.8]	2.2 [1.8, 2.6]	<0.001
Postcomplications	20 (12.82)	8 (8.51)	0.289	20 (12.4)	9 (10.0)	0.558
PPCs	19 (12.18)	7 (7.45)	0.330	19 (11.8)	8 (8.9)	0.509
Gastroparesis	5 (3.21)	0 (0.00)	0.380	5 (3.1)	0 (0.0)	0.399
Anastomotic fistula	2 (1.28)	2 (2.13)	0.693	2 (1.2)	1 (1.1)	0.628
Bleeding	2 (1.28)	0 (0.00)	0.320	4 (2.5)	3 (3.3)	0.294
Hospitalization cost (CNY)	89,614 [76,778, 97,986]	83,963 [72,476, 94,814]	0.065	87,869 [74,123, 97,931]	85,361 [72,487, 94,936]	0.624
Length of stays (days)	10.00 [9.00, 12.00]	11.00 [9.00, 12.00]	0.675	10.0 [9.0, 12.0]	11.0 [9.0, 12.0]	0.243
Long-Term Outcomes						
3-year RFS	75.20%	79.12%		78.86%	78.00%	
Cox analysis	HR = 0.92, 95% CI (0.51–1.65)		0.392	HR = 1.14, 95% CI (0.55–2.35)		0.721
3-year OS	74.16%	82.19%		78.17%	81.48%	
Cox analysis	HR = 0.75, 95% CI (0.39–1.44)		0.770	HR = 0.98, 95% CI (0.42–2.27)		0.955

*LN, lymph node; Pain scores, the average scores of the 11-point (0–10) numerical rating scales 5 days after surgery; PPCs, postoperative pulmonary complications; TLG, totally laparoscopic gastrectomy; LAG, laparoscopic-assisted gastrectomy; PS-IPTW, propensity score-based inverse probability of treatment weighting; RFS, relapse-free survival; OS, overall survival; HR, hazard ratio; CI, confidence interval*.

In terms of short-term outcomes, the gastrointestinal function recovery of TLG, which included the median time of the first flatus, and first defecation were about 3 and 4 days, respectively. The median times of first drinking water, first liquid food, and removal of the nasogastric tube were 5, 8, and 5 days, respectively. According to Clavien–Dindo classification, the most common postoperative complications were postoperative pulmonary complications (PPCs) (8.9%). The median hospitalization cost was 85,361 (IQR 72,487, 94,936) CNY, and the median length of stay was 11 (9, 12) days. Among them, TLG showed that its short-term outcomes were not significantly different from those of LAG (*p* > 0.05). Although TLG showed a benefit in reducing wound discomfort, which the median pain score was 0.4 points lower than LAG (LAG vs. TLG: 2.6 vs. 2.2, *p* < 0.001).

**Figure 1:**
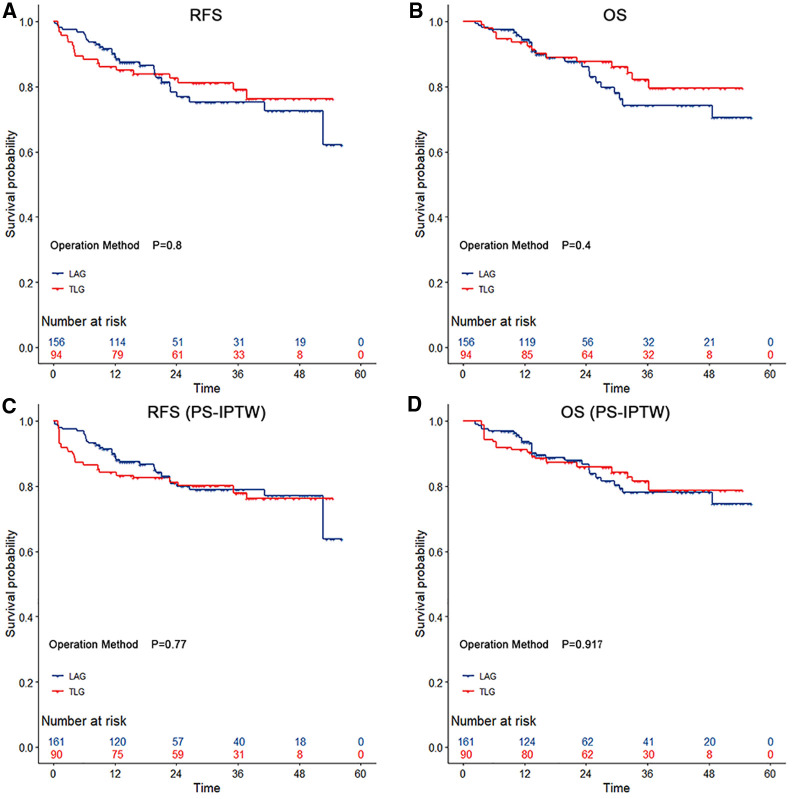
The survival curves among gastric cancer with operation methods during the propensity score-based inverse probability of treatment weighting. According to the type of surgery, both totally laparoscopic gastrectomy and laparoscopic-assisted gastrectomy showed no significant differencess in relapse-free survival and overall survival (*p* > 0.05 by the log-rank test). The risk tables show the actual number of patients with operation methods.

In terms of long-term outcomes, all 250 patients had completed follow-up by September 2021, and the median follow-up time was 25.1 (IQR 21.3–29.0) months. During the follow-up period, 48 patients relapsed after surgery, and 39 died. After the PS-IPTW, there were no significant differences between groups in the 3-year RFS rate (LAG vs. TLG: 78.86% vs. 78.00%; HR = 1.14, 95% CI, 0.55–2.35; *p* = 0.721) and the 3-year OS rate (LAG vs. TLG: 78.17% vs. 81.48%; HR = 0.98, 95% CI, 0.42–2.27; *p* = 0.955). Figure 1 depicts the Kaplan–Meier survival curves and log-rank tests, showing that TLG has similar survival outcomes to LAG.

### Subgroup Analysis

**Figure 2:**
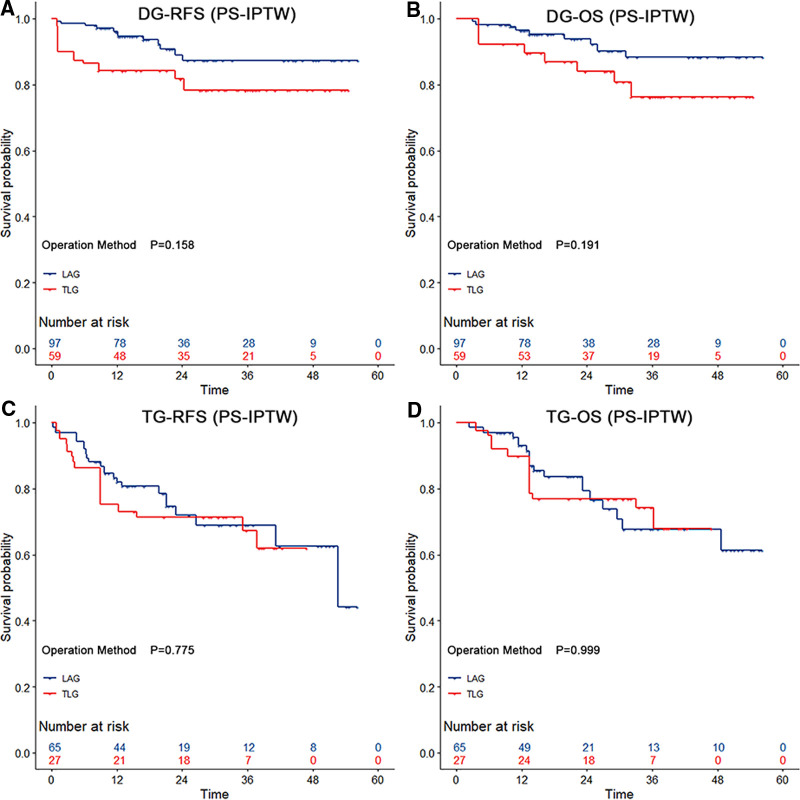
The survival curves among distal and total gastrectomy after the propensity score-based inverse probability of treatment weighting. Both distal gastrectomy and total gastrectomy showed no significant difference in relapse-free survival and overall survival (*p* > 0.05 by the log-rank test). The risk tables show the actual number of patients with operation methods.

By using the same PS-IPTW procedures to balance the between-group disparities, except for the dissected numbers of lymph nodes, similar prognosis outcomes could be found in the subgroup analysis of LADG and TLDG (Table 5). However, as compared to LATG, TLTG did not increase the operation time (*p* = 0.216), and the wound pain scores did not indicate a significant advantage (*p* = 0.126). The 3-year RFS rate (LADG vs. TLDG: 87.32% vs. 78.26%; HR = 2.19, 95% CI, 0.69–6.92; *p* = 0.182) and the 3-year OS rate (LADG vs. TLDG: 88.23% vs. 76.25%; HR = 2.21, 95% CI, 0.64–7.57; *p* = 0.209) showed no significant difference in distal gastrectomy. The 3-year RFS rate (LATG vs. TLDG: 68.96% vs. 67.20%; HR = 1.19, 95% CI, 0.46–3.08; *p* = 0.716) and the 3-year OS rate (LATG vs. TLTG: 67.63% vs. 74.30%; HR = 1.03, 95% CI, 0.34–3.12; *p* = 0.959) also showed no significant difference in total gastrectomy. Figure 2 demonstrates that TLG has comparable survival outcomes to LAG in both distal and total gastrectomy.

**Table 5:** Subgroup analysis of operation methods after PS-IPTW.

Characteristics	Distal Gastrectomy (*N* = 156)			Total Gastrectomy (*N* = 92)		
	LADG (*N* = 97)	TLDG (*n* = 59)	*p*	LATG (*n* = 65)	TLTG (*n* = 27)	*p*
Operative Outcomes						
Operation time (minutes)	240.0 [195.0, 278.3]	270.0 [240.0, 300.0]	0.001	265.0 [240.0, 289.1]	270.0 [240.0, 316.4]	0.216
Blood loss (ml)	50.0 [40.0, 74.2]	30.0 [25.0, 40.0]	<0.001	60.0 [50.0, 100.0]	30.0 [30.0, 50.0]	<0.001
Positive LN	0.0 [0.0, 3.0]	1.0 [0.0, 4.2]	0.338	2.0 [0.0, 10.3]	3.6 [0.0, 16.0]	0.435
Dissected LN	28.0 [21.0, 32.0]	28.9 [24.0, 39.9]	0.089	25.0 [21.1, 38.2]	34.6 [23.6, 43.6]	0.195
Transfusion						
No	93 (95.9)	50 (84.7)	0.100	51 (78.5)	26 (96.3)	0.084
Yes	5 (5.2)	9 (15.3)		14 (21.5)	2 (7.4)	
Short-Term Outcomes						
First flatus (days)	3.0 [3.0, 3.0]	3.0 [2.0, 3.0]	0.226	3.0 [3.0, 4.0]	3.0 [2.0, 4.0]	0.362
First defecation (days)	4.0 [3.0, 5.0]	4.0 [3.3, 5.0]	0.739	5.0 [4.0, 6.0]	5.0 [3.5, 6.0]	0.908
First drinking water (days)	5.0 [4.0, 6.0]	5.0 [4.0, 5.5]	0.147	6.0 [5.0, 6.0]	6.0 [5.0, 7.0]	0.052
First liquid food (days)	7.0 [6.0, 8.0]	7.0 [6.0, 8.0]	0.824	8.0 [7.0, 9.0]	8.9 [7.0, 9.0]	0.102
Nasogastric tube (days)	4.0 [3.0, 6.0]	4.0 [3.0, 6.0]	0.935	6.0 [4.0, 8.0]	5.0 [5.0, 7.0]	0.911
Pain scores (points)	2.6 [2.0, 2.8]	2.2 [1.8, 2.6]	0.012	2.8 [2.6, 2.8]	2.44 [2.2, 2.8]	0.126
Postcomplications	8 (8.2)	3 (5.1)	0.344	10 (15.4)	2 (7.4)	0.368
PPCs	7 (7.2)	2 (3.4)	0.234	10 (15.4)	2 (7.4)	0.368
Gastroparesis	4 (4.1)	0 (0.0)	0.300	1 (1.5)	0 (0.0)	0.528
Anastomotic fistula	1 (1.0)	1 (1.7)	0.385	1 (1.5)	0 (0.0)	0.527
Bleeding	3 (3.0)	1 (1.7)	0.447	1 (1.5)	3 (11.1)	0.129
Hospitalization cost (CNY)	85,118 [68,384, 97,922]	84,167 [69,566, 92,822]	0.800	92,435 [79,281, 97,840]	85,141 [76,809, 91,180]	0.152
Length of stays (days)	10.0 [9.0, 11.0]	10.0 [9.0, 11.0]	0.628	11.4 [10.0, 13.00]	12.0 [11.1, 14.0]	0.079
Long-Term Outcomes						
3-year RFS	87.32%	78.26%		68.96%	67.20%	
Cox analysis	HR = 2.19, 95% CI (0.69–6.92)		0.182	HR = 1.19, 95% CI (0.46–3.08)		0.716
3-year OS	88.23%	76.25%		67.63%	74.30%	
Cox analysis	HR = 2.21, 95% CI (0.64–7.57)		0.209	HR = 1.03, 95% CI (0.34–3.12)		0.959

*RFS, relapse-free survival; OS, overall survival; PS-IPTW, propensity score-based inverse probability of treatment weighting; LADG, laparoscopic-assisted distal gastrectomy; TLDG, totally laparoscopic distal gastrectomy; LATG, laparoscopic-assisted total gastrectomy; TLTG, totally laparoscopic total gastrectomy; LN, lymph node; PPCs, postoperative pulmonary complications; HR, hazard ratio; CI, confidence interval*.

## Discussion

The usefulness and effectiveness of intracorporeal vs. extracorporeal approaches in a variety of surgical disciplines are currently a matter of dispute. Many studies have shown that in early or locally advanced gastric cancer, the long-term result of laparoscopic gastrectomy is comparable to that of open gastrectomy (24, 25). A majority of laparoscopic procedures are LADG, and large-scale prospective studies of TLG are still lacking.

This study compared the short- and long-term prognoses of gastric cancer patients who had LAG and TLG. A total of 250 GC patients were included in the study. After using the PS-IPTW to balance the baseline and pathological features of the TLG and LAG groups, we found that TLG took a longer operation time than LAG (*p* < 0.05) but resulted in more lymph nodes retrieved, less blood loss, and less wound discomfort (*p* < 0.05). Furthermore, there was no signiﬁcant difference in long-term prognosis between the two groups (*p* > 0.05).

For TLDG, Jin et al. (26) reported that a meta-analysis of 25 studies involving 4,562 gastric cancer patients revealed that postoperative complications were comparable for TLDG and LADG. However, TLDG had favorable short-term results such as blood loss, time of liquid feed, and hospital stay (*p* < 0.05). Besides, Milone et al. (27) reported that a meta-analysis of 3,818 gastric cancer patients under distal gastrectomy showed that the less intraoperative blood loss, the more the harvested lymph nodes and the shorter the length of hospital stay in TLDG than in LADG (*p* < 0.05). Our study also showed similar results in TLDG. We found that this similarity may be due to the fact that intracorporeal reconstruction proved difficult, and TLDG took 30 min longer operation time than LADG, but there did not seem to be an increased risk of postoperative complications. Despite the longer operation duration, TLG showed benefits in terms of decreased intraoperative blood loss and wound pain, as well as a greater number of lymph node dissections (*p* < 0.05), without increasing hospital stay or costs (*p* > 0.05). The possible reasons for these might be that the intracorporeal approaches minimize inadequate surgical field exposure, severe anastomotic tugging, and bleeding produced by laparoscopically assisted small incisions. Besides, the lymph node tracking technologies may result in a more dissected number of lymph nodes. While possibly due to the conservative treatment strategies, the small length of the abdominal incision and pain response of TLG patients did not result in a significant advantage in gastrointestinal function recovery.

Umemura et al. (28) completed a review paper that covered 25 articles on TLTG and demonstrated that it tended to consume more surgical time while having advantages in terms of intraoperative blood loss and postoperative recovery. However, Milone et al. (27) revealed that TLTG was not statistically different from LATG for the above-mentioned outcomes. Our study also showed similar results for TLTG. TLTG revealed no significant difference in prognosis outcomes compared with LATG (*p* > 0.05), except for blood loss (LATG vs. TLTG: 50 ml vs. 30 ml, *p* < 0.001). A clearer vision of intracorporeal approaches, particularly in esophageal exposure and esophagojejunal anastomosis, could explain why the operation time of TLTG is not longer than that of TLAG. Furthermore, our research found that TLG had advantages in the sense that less intraoperative traction can prevent subsequent injury caused by excessive traction of the residual stomach, esophagus, and other tissues. This was also more consistent with the principle of a tumor-free operation in which the excision specimens were intracorporeally put into the bag, which could prevent their appearance in the tumor tissue of extrusion.

Studies (29, 30) showed that the occurrence of complications was not determined by the totally laparoscopic approach. Our study also confirmed this, as the Chi-square test revealed no significant difference between the two groups. All 94 TLG patients completed the R0 resection, including 62 TLDG patients (66.0%) and 32 TLTG patients (34.0%), of which only 8 patients (8.51%) suffered from postoperative complications, including 7 cases of PPCs (7.45%) and 2 cases of anastomotic fistula (2.13%) (One patient developed both complications). No gastroparesis and postoperative bleeding occurred in the TLG group. Once the postoperative complications occurred, the same treatments were given in both surgery groups, including conservative and special treatments. Conservative treatments included atomizing, expectorant drugs, antibiotic therapy, dietary abstinence, gastric tube drainage, abdominal drainage, or abdominal double-cannula lavage. Special treatments included chest drainage, trachea cannula, a second surgery, or intensive care. The aforementioned two of seven PPC patients who had respiratory failure were admitted to the Intensive Care Unit (ICU) and treated with a trachea cannula, anti-infection, and other Advanced Cardiac Life Support measures. The two patients with anastomotic fistula were diagnosed with small fistula and were treated with dietary abstinence, anti-infection, continuous gastric tube, and abdominal drainage.

Following the PS-IPTW in our study, the multivariate Cox regression analysis revealed that the pN stage was an independent risk factor for the recurrence and mortality of laparoscopic surgery, regardless of the operation method. Favorable long-term outcomes have been reported in the limited number of studies comparing LAG with TLG. Moisan Fabrizio et al. (31) reported in a matched cohort study of 31 patients of both open and TLG groups that the 3-year RFS rate and the 3-year OS rate were 79.4% and 82.3%, respectively. Besides, the survival outcomes also showed similar survival rates of LAG and TLG after the PS-IPTW, in which RFS and OS were 78.86% vs. 78.00% and 78.17% vs. 81.48%, respectively. Similar results could be found in the subgroup analysis. TLG did not increase the survival risks in long-term outcomes.

The limitation in our research was that it was a retrospective study, which meant that the treatment strategies were not determined by random assignments, and, therefore, selection bias may have occurred even when using the groups’ balanced method of the PS-IPTW. Secondly, except for the reverse puncture device reconstruction, our surgical team also attempted to perform other intracorporeal endoscopic anastomoses such as overlap (32), isoperistaltic jejunum-later-cut overlap (33), or π-shaped esophagojejunal anastomoses (34) during the study period. Although no serious postoperative complications occurred in these operations, the small number of these surgeries may have resulted in confounding bias, and, therefore, they were not included in this analysis. Besides, because the survival rates in both groups were comparable, the other survival outcomes that were lacking in this study might more substantially guide decisions on the manner of operation.

In conclusion, minimally invasive treatment is a major trend in surgical development (35). However, TLG should be based on the surgeon’s technical skills, the patient’s physical condition, objective economic status, and the features of the equipment used. The following are some of our study’s recommendations: (1) We could endoscopically inject carbon nanoparticles or ICG suspension around the tumor 1 day before TLG to identify the tumor boundaries. (2) For Billroth-II with Braun anastomosis in TLDG, the input loop should not be too long, and the mesenterium should not be twisted. (3) For the reverse puncture device reconstruction in TLTG, place the anvil of the esophageal stump first and then cut the esophagus. It is easy to place the anvil under esophageal traction; (4) Choose a smooth needle thread with high tension resistance, with an appropriate length of around 10 cm, and continuously reinforce the anastomosis under laparoscopy. (5) All should follow the same fundamental principles as an open radical gastrectomy. In case of severe complications that are difficult to manage under laparoscopy, we should switch over to laparotomy. Elaborate considerations should be made to maximize the benefits accrued to patients.

This study showed that TLG for stomach cancer is safe and feasible in both short- and long-term prognoses. Although the surgical procedure is tough to perform, it necessitates greater expertise and coordination on the part of the surgeon. The current long-term efficacy of totally laparoscopic radical gastrectomy still needs evidence-based medical confirmation in the form of large randomized controlled trials.

## Data Availability

The raw data supporting the conclusions of this article will be made available by the authors, without undue reservation.
